# The Role of Human Platelet Preparation for Toll-Like Receptors 2 and 4 Related Platelet Responsiveness

**DOI:** 10.1055/s-0039-1685495

**Published:** 2019-04-17

**Authors:** Juergen Koessler, Marius Niklaus, Katja Weber, Angela Koessler, Sabine Kuhn, Markus Boeck, Anna Kobsar

**Affiliations:** 1 Institute of Clinical Transfusion Medicine and Haemotherapy, University of Würzburg, Würzburg, Germany

**Keywords:** toll-like receptor, lipopolysaccharides, chemokine, platelet preparation

## Abstract

**Background** Like immune cells, platelets express the repertoire of toll-like receptors (TLR), among them TLR2 and TLR4, which are important for the recognition of bacterial patterns. Receptor-mediated functional effects in platelets have been investigated, but reliable conclusions are tampered due to heterogeneous study designs with variable platelet preparation methods. This study compares TLR2- and TLR4-dependent platelet responsiveness in platelet-rich plasma (PRP) and in washed platelets (WPs).

**Material and Methods** Fresh peripheral blood samples from healthy donors served for the preparation of PRP and WP. Basal and agonist-stimulated TLR2 and TLR4 expression levels were evaluated by flow cytometry. Light transmission aggregometry was used to investigate functional effects of TLR2 and TLR4 stimulation with Pam3CSK4 or LPS (lipopolysaccharides from *Escherichia coli* ) as ligands. The capacity of chemokine release was determined by immunoassays.

**Results** Pam3CSK4 and LPS (in combination with thrombin) were able to induce aggregation in WP, but not in PRP, with threshold concentrations of 15 µg/mL. Basal expression levels of TLR2 and TLR4 were higher in WP than in PRP, increasing several-fold rapidly and persistently upon platelet activation with potent agonists. Pam3CSK4 (15 µg/mL) or LPS led to the submaximal release of RANTES, PF4, PDGF, NAP-2, and sCD40L from WP. In PRP, secretory effects are less pronounced for RANTES, PDGF, or PF4, and not detectable for NAP-2 or sCD40L.

**Conclusion** The effects mediated by TLR2 and TLR4 stimulation are dependent on platelet preparation, an important issue for experimental designs and for manufacturing of platelet concentrates in transfusion medicine.

## Introduction

Platelets are small nonnucleated cells that are involved in the processes of hemostasis and thrombosis. However, there is growing evidence that platelets play an essential role in mechanisms of inflammation and in immunological reactions. 1

Circulating platelets are among the first cells being confronted with foreign particles at the site of injuries. Platelets themselves have the ability to migrate into tissues and to interact with lymphocytes, dendritic cells, or macrophages modulating their function. 2 3 Like immune cells or other cell types (e.g., endothelial cells, epithelial cells), platelets express different toll-like receptors (TLRs), among them TLR2 and TLR4, permitting the direct recognition of pathogen-associated molecular patterns (e.g., various bacterial antigens). 1 4 5 6

TLR2 is able to recognize components of gram-positive bacteria and to exert effects on platelet signal transduction, aggregation, adhesion, or on platelet–neutrophil interactions. 7 8 9 10 11 The synthetic ligand Pam3Cys-Ser-(Lys)4 (Pam3CSK4), a triacylated peptide, has frequently been used for platelet TLR2 stimulation in experimental studies. 7 8 Lipopolysaccharide (LPS) is an outer membrane component of gram-negative bacteria and a potent mediator of bacterial sepsis. Platelet TLR4 is able to bind LPS, thereby initiating platelet activation and also interactions with neutrophils. 12 13 Although their presence on platelets has been confirmed in several studies, 1 4 5 6 10 12 14 the regulation of TLR2 and TLR4 expression during platelet activation has not been analyzed comparatively in different platelet sources.

In addition, results of previous studies addressing the role of TLR2 and TLR4 for the initiation of platelet aggregation were conflicting. 8 15 In washed platelets (WPs), aggregation responses were inducible with Pam3CSK4 7 8 9 15 or potentiated with LPS in combination with low-dose thrombin, 15 but not in a study using platelet-rich plasma (PRP). 16

Moreover, the stimulation of TLR2 and TLR4 with bacterial ligands has been shown to result in the activation of platelet signaling cascades accompanied by the shedding of contents. 8 17 In this regard, differences in the extent of chemokine releases have been observed. Pam3CSK4 and LPS stimulation led to the release of von Willebrand factor, as α-granule marker, from WP. 15 Instead, TLR4 stimulation did not support shedding of PDGF (platelet-derived growth factor), RANTES ( *r* egulated on *a* ctivation, *n* ormal *T* cell *e* xpressed and *s* ecreted), or PF4 (platelet factor 4) in PRP. 18

This study, therefore, compared TLR2- and TLR4-dependent platelet responsiveness in PRP and in WP, as commonly used preparation methods for platelet research. 19 For the evaluation of functional effects, aggregation studies were performed to obtain threshold concentrations for the TLR ligands initiating aggregation responses. Based on these findings, the quantity of chemokine release was analyzed in WP and in PRP to estimate the capacity of TLR2 and TLR4 stimulation for the release of PF4, PDGF, RANTES, CD40L, and NAP-2 as important chemokines from α-granules.

## Materials and Methods

### Materials

Adenosine diphosphate (ADP) and thrombin receptor activating peptide-6 (TRAP-6) were from Haemochrom Diagnostica GmbH (Essen, Germany). Convulxin was obtained from Enzo Life Sciences GmbH (Lörrach, Germany), and Pam3CSK4 (VacciGrade, synthetic triacylated lipopeptide; endotoxin level <0.05 EU/µg, purity ≥ 95% [UHPLC]) from InvivoGen (Toulouse, France). Ethylene glycol-bis(β-aminoethylether)-N,N,N',N'-tetraacetic acid (EGTA), prostaglandin E1 (PGE1), epinephrine, LPS from *Escherichia coli* O111:B4 (purified by ion-exchange chromatography; impurities ≤1% protein, ≤1% RNA; TLR ligand tested), thrombin, fibrinogen (from human plasma), fetal bovine serum, Tyrode's salt solution, mouse IgG2a (isotype), FITC-conjugated goat antimouse polyclonal antibody, and NAP-2 ELISA Kit were from Sigma-Aldrich Chemie GmbH (München, Germany). Mouse monoclonal anti-TLR2 (clone TL2.1, recognizing a TLR2-associated epitope) 20 and anti-TLR4 antibodies (clone HT125 recognizing the N-terminal domain of TLR4) 21 were from Thermo Fisher Scientific (Darmstadt, Germany). FITC-conjugated mouse anti-CD62P and an appropriate FITC-conjugated isotype control were from OriGene Technologies GmbH (Herford, Germany). FITC-conjugated mouse antifibrinogen antibody and an appropriate FITC-conjugated isotype control were from BioCytex SARL (Marseille, France). RANTES, PF4, sCD40L, and PDGF ELISA Kits were from R&D Systems GmbH (Wiesbaden-Nordenstadt, Germany).

### Blood Collection and Platelet Preparation

Our studies with human platelets and the consent procedure were approved by the local ethics committee of the University of Würzburg (approval number 101/15). All participants provided their written informed consent. The study was performed according to our institutional guidelines and to the declaration of Helsinki.

Peripheral blood samples from informed healthy voluntary donors (aged from 21 to 49 years, without any medication 14 days before donation) were collected in polystyrene tubes containing 3.2% citrate buffer (106 mM trisodium citrate; Sarstedt, Nuembrecht, Germany). PRP was obtained by centrifugation of blood samples at 280 × *g* for 5 minutes. For the preparation of WP, 3 mM EGTA was added to whole blood to prevent platelet activation. Only the upper 75% of the PRP supernatant was used for experiments to minimize leukocyte contamination with leukocyte concentrations below the detection limit of 0.1 × 10 ^3^ /µL in the blood cell count.

After that, 75 nM PGE1 was added to PRP, and platelets were pelleted at 430 × *g* for 10 minutes. The pelleted platelets were washed once in CGS buffer (120 mM sodium chloride, 12.9 mM trisodium citrate, 30 mM D-glucose, pH 6.5) containing 75 nM PGE1, and resuspended in Tyrode's salt solution.

Platelet concentrations in PRP and WP were equally adjusted to platelet concentrations of 3.5 × 10 ^8^ /mL. Blood cell counts were measured with the hematology analyzer KX21N from Sysmex GmbH (Norderstedt, Germany).

### Platelet Aggregation

Light transmission aggregation was measured using an APACT 4004 aggregometer (LabiTec, Ahrensburg, Germany); 200 µL of PRP or WP suspension supplemented with 1 mM CaCl _2_ was stimulated with Pam3CSK4 diluted in PBS with 0.01% FCS or with LPS. Aggregation was measured for 5 minutes under continuous stirring at 1,000 rpm and 37°C.

In LPS studies, samples were costimulated with threshold concentrations (as the lowest concentration leading to submaximal aggregation responses) of 0.025 U/mL thrombin for WP and of 1 µM TRAP-6 for PRP. Since the “natural” agonist thrombin is not appropriate for aggregation and flow cytometry studies in PRP due to fibrinogen cleavage and clot formation, TRAP-6 was used.

Stimulations with 10 µM TRAP-6 or 0.5 U/mL thrombin served as controls inducing stable aggregation.

### Measurement of TLR2 and TLR4 Expression by Flow Cytometry

For the measurement of TLR2 and TLR4 expression, 40 µL of PRP was stained with 3 µL of anti-TLR2 or anti-TLR4 antibodies or isotype control for 10 minutes at 37°C. For WP, 1 mM CaCl _2_ was added to platelet suspensions shortly before stimulation. After that, 40 µL of WP was stained with 3 µL of mouse anti-TLR2 or anti-TLR4 antibodies or isotype control for 10 minutes at 37°C. The samples (PRP or WP) were stimulated for 2 to 30 minutes at 37°C with buffer (control), 10 µM TRAP-6, 10 µg/mL convulxin, 10 µM ADP, or with 8 µM epinephrine. Samples were stopped with 1% formaldehyde (final concentration), fixed for 10 minutes at room temperature, and centrifuged for 1 minute at 14,000 × *g* . The pellet was resuspended in 100 µL of PBS/BSA/Glc (Dulbecco's PBS/Ca ^2+^ , Mg ^2+^ free, 5.5 mM D-glucose, 0.5% BSA) and stained at room temperature in the dark for 30 minutes with 1 µM of FITC-conjugated goat antimouse antibody. Then, samples were diluted with 300 µL of PBS/BSA/Glc and analyzed by flow cytometry with the FACSCalibur flow cytometer from Becton Dickinson (Franklin Lakes, New Jersey, United States) using CELLQuest software, version 6.0. The platelet population was identified by its forward and side scatter distribution and *2* 0,000 events were analyzed for mean fluorescence.

### Measurement of CD62P Expression and Fibrinogen Binding by Flow Cytometry

For the measurement of CD62P expression, 30 µL of PRP or WP was stained with 3 µL of FITC-conjugated mouse anti-CD62P antibody or isotype control for 10 minutes at 37°C. For WP, 1 mM CaCl _2_ was added to platelet suspensions before stimulation. After that, samples were stimulated with buffer (basal expression) or 10 µM TRAP-6 for 2 minutes at 37°C, followed by fixation with 1% formaldehyde (final concentration) for 10 minutes at room temperature in the dark. Then, samples were diluted with 500 µL of PBS/BSA/Glc and analyzed by flow cytometry as described earlier.

For the measurement of fibrinogen binding, WP was supplemented with fibrinogen (100 µg/mL final concentration). Then 15 µL of PRP or of fibrinogen-supplemented WP was stained with 15 µL of FITC-conjugated mouse antifibrinogen antibody or isotype control for 10 minutes at 37°C. For WP, 1 mM CaCl _2_ was added to platelet suspensions before stimulation. After that, samples were stimulated with buffer (basal fibrinogen binding) or 10 µM TRAP-6 for 2 minutes at 37°C, followed by fixation with 1% formaldehyde (final concentration) for 10 minutes at room temperature in the dark. Then, samples were diluted with 500 µL of PBS/BSA/Glc and analyzed by flow cytometry as described earlier.

### Measurement of Chemokine Levels

For the measurement of chemokine levels, 220 µL of PRP or WP suspension supplemented with 1 mM CaCl _2_ was stimulated with buffer (control), 15 µg/mL Pam3CSK4 diluted in PBS with 0.01% FCS, 15 µg/mL LPS, 10 µM TRAP-6, or 0.5 U/mL thrombin (positive control) for 30 minutes at room temperature. After that, samples were centrifuged for 1 minute at 14,000 × *g* and supernatants were divided into five tubes and stored at −80°C. The concentrations of released RANTES, PF4, sCD40L, PDGF, and NAP-2 were measured by corresponding immunoassay kits.

### Statistical Analysis

Data are presented as mean ± standard error of the mean (SEM). The *n* -values refer to the number of experiments, each made with different blood donors. Data were analyzed by ANOVA using GraphPad PRISM 7 (GraphPad Software, San Diego, California, United States) and MedCalc statistic program (MedCalc Software bvba, Mariakerke, Belgium). *p* < 0.05 was considered statistically significant.

## Results

### The TLR2 Ligand Pam3CSK4 and the TLR4 Ligand LPS Have the Capacity to Promote Platelet Aggregation in WP, but not in PRP

**Fig. 1:**
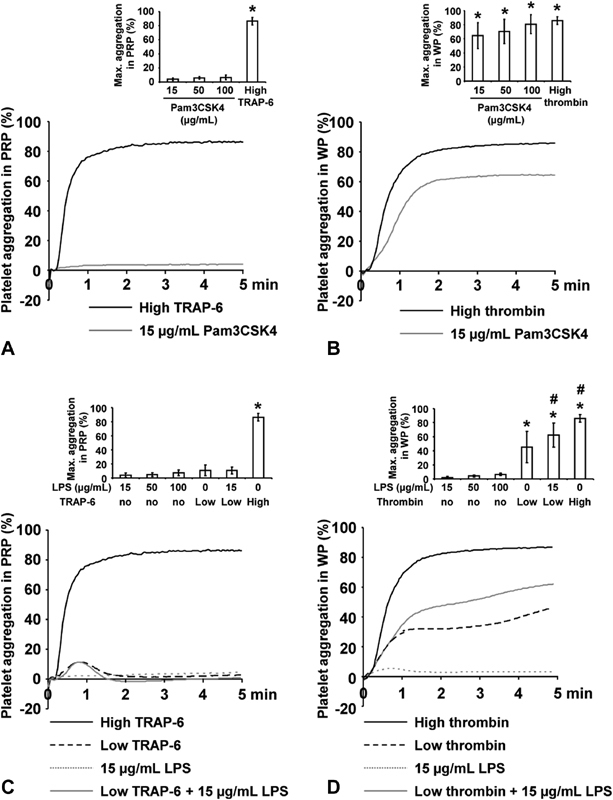
The effect of Pam3CSK4 and LPS on platelet aggregation. Mean traces of light transmission aggregometry are shown, and in addition as bar graphs, corresponding maximal aggregation values as mean ± SD. ( **A** ) PRP was stimulated with 15 µg/mL Pam3CSK4 or with 10 µM TRAP-6 as control. ( **B** ) WP was stimulated with 15 µg/mL Pam3CSK4 or with 0.5 U/mL thrombin as control. ( **C** ) PRP was stimulated with 15 µg/mL LPS, with a low 1 µM TRAP-6 concentration alone or in combination with 15 µg/mL LPS. TRAP-6 in a high 10 µM concentration served as control. ( **D** ) WP was stimulated with 15 µg/mL LPS, with a low 0.025 U/mL thrombin concentration or with 0.025 U/mL thrombin in combination with 15 µg/mL LPS. The high 0.5 U/mL thrombin concentration served as control; *n* = 10; * *p* < 0.05 compared with unstimulated samples; # *p* < 0.05 compared with samples stimulated with low thrombin concentration.

The role of TLR2 and TLR4 for the activation of platelets was analyzed by transmission light aggregometry ( Fig. 1 ) using the synthetic peptide Pam3CSK4, as TLR2 agonist, and LPS from *E. coli* , as TLR4 agonist.

Stimulation of PRP with Pam3CSK4 up to 100 µg/mL did not induce aggregation ( Fig. 1A ), whereas freshly prepared WP was able to aggregate upon TLR2 stimulation with 15 µg/mL Pam3CSK4 or higher concentrations ( Fig. 1B ).

TLR4 stimulation with LPS up to 100 µg/mL did not induce aggregation in PRP ( Fig. 1C ). Similarly, LPS alone was not able to trigger platelet aggregation in WP ( Fig. 1D ). However, in WP costimulated with a threshold concentration of thrombin (0.025 U/mL), aggregation was promoted with 15 µg/mL LPS. Instead, 15 µg/mL LPS did not mediate supporting effects on aggregation in PRP, costimulated with the threshold concentration of 1 µM TRAP-6.

### TLR2 and TLR4 Expression Is Higher in WP than in PRP and Depends on the Degree of Platelet Activation

**Fig. 2:**
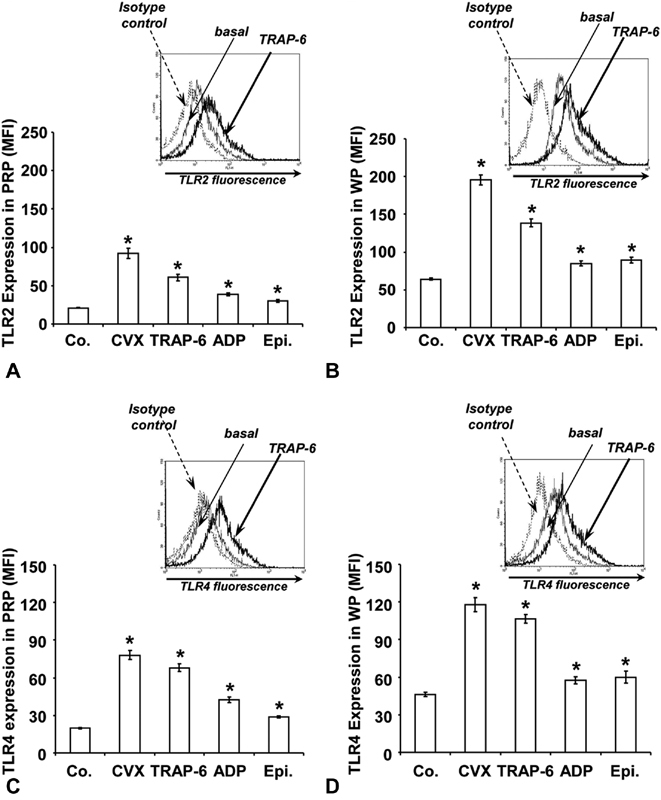
TLR2 and TLR4 expression stimulated by different agonists. The histograms show the mean fluorescence intensity (MFI) of TLR2 and TLR4 surface expression in PRP ( **A** and **C** ) and in WP ( **B** and **D** ) stimulated for 2 minutes with buffer as control (Co.), with 10 µg/mL convulxin (CVX), 10 µM TRAP-6, 10 µM ADP, or 8 µM epinephrine (EPI). Results are presented in absolute arbitrary units as mean ± SEM; *n* = 10; * *p* < 0.05 compared with controls.

The expression of TLR2 and TLR4 on the platelet surface was investigated by flow cytometry using receptor-specific antibodies. The extent of induced expression was analyzed with various platelet activators ( Fig. 2 ).

The basal receptor levels were higher on WP than in PRP. A selective and potent agonist of the platelet collagen receptor glycoprotein VI, 10 µg/mL convulxin increased the expression levels several fold for both receptors in PRP and on WP ( Fig. 2A–D ). Similar effects were induced by 10 µM TRAP-6.

ADP (10 µM) as weak agonist stimulated TLR2 and TLR4 expression in PRP approximately by twofold ( Fig. 2A, B ) and less emphasized on WP ( Fig. 2C, D ). Incubation with 8 µM epinephrine as another weak agonist slightly increased TLR2 and TLR4 expression in PRP and on WP.

### Platelet Activation Induces a Rapid and Persistent Upregulation of TLR2 and TLR4 Expression in Both Preparations

**Fig. 3:**
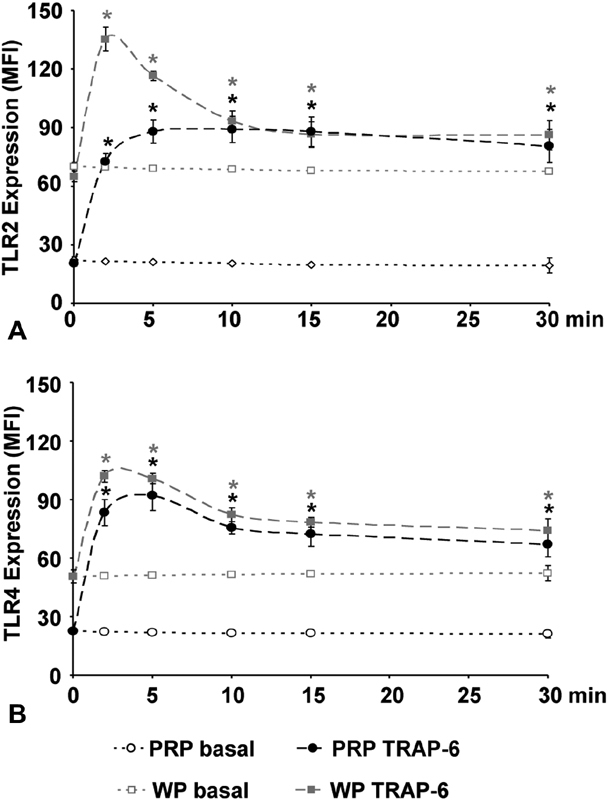
Time-dependent TLR2 and TLR4 expression under TRAP-6 stimulation. The mean fluorescence intensity (MFI) of basal TLR2 ( **A** ) and TLR4 ( **B** ) expression in PRP (open circles), in WP (open squares), or of 10 µM TRAP-6 stimulated TLR2 and TLR4 expression in PRP (black circles) and in WP (black squares) is shown. The data are presented as mean ± SEM; *n* = 10; * (dark): *p* < 0.05 compared with basal values at the same time point; * (bright): *p* < 0.05 compared with 0 minutes.

Since TRAP-6 induced effects were significant for TLR2 and TLR4 in both milieus, we selected TRAP-6 for kinetic studies ( Fig. 3 ). Basal TLR2 and TLR4 expression remained stable for the observed period of 30 minutes. In WP, the levels were significantly higher than in PRP, approximately threefold for TLR2 ( Fig. 3A ) and twofold for TLR4 ( Fig. 3B ). The stimulation of platelet thrombin receptor PAR-1 with 10 µM TRAP-6 resulted in an almost threefold increase of both TLR2 and TLR4 expression in PRP after 2 minutes. The expression on WP showed a twofold increase.

TLR2 expression increased to its maximum within 2 minutes after TRAP-6 stimulation on WP and within 5 minutes in PRP. Once activated, TLR2 expression in PRP remained unchanged for the next 30 minutes, whereas it declined on WP within the next 10 minutes, similar to PRP ( Fig. 3A ). The peak of TLR4 expression was also reached faster in WP than in PRP (5 vs. 2 minutes), followed by a slight decline within the next 10 minutes to stable levels for the consecutive period ( Fig. 3B ).

### The Released Chemokine Levels Induced by the TLR2 Ligand Pam3CSK4 and the TLR4 Ligand LPS Are more Pronounced in WP than in PRP

Upon activation, platelets are able to shed chemokines from the α-granules like sCD40L, 1 which is involved in platelet–leucocyte interactions and in the functional modulation of immune cells. 22 PDGF is dedicated to be a potent growth factor, to act on vascular smooth muscle cells, and to promote arteriosclerosis. 23 PF4 (platelet factor 4) is one of the first identified platelet cytokines and a strong chemoattractant for neutrophils. 24 It regulates the function of monocytes 25 and the cytokine release from activated T-cells. 26 NAP-2 is another cytokine with high levels in platelets, attributed to play a significant role for neutrophil recruitment in response to vascular injury. 27 RANTES represents a chemotactic mediator for monocytes, eosinophils, and T-cells triggering cytokine synthesis in key effector cells. 28

**Fig. 4:**
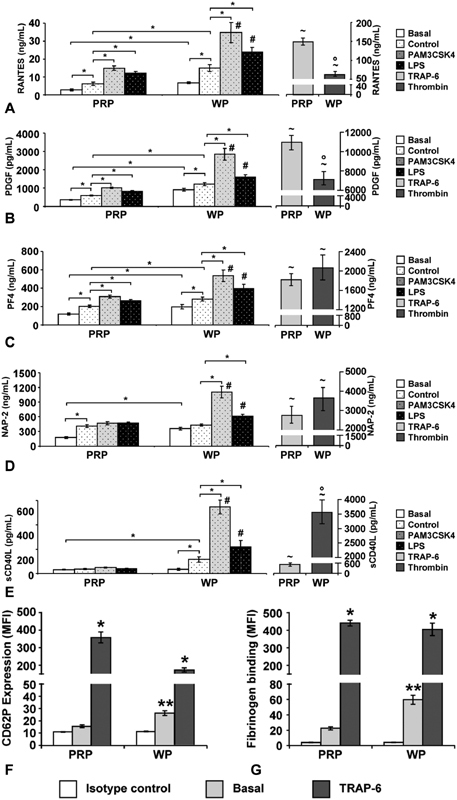
Chemokine levels in WP and PRP under Pam3CSK4 and LPS stimulation, and CD62P expression and fibrinogen binding after preparation. ( **A** – **E** ) The concentrations of RANTES ( **A** ), PDGF ( **B** ), PF4 ( **C** ), NAP-2 ( **D** ), and sCD40L ( **E** ) were measured in PRP or in the supernatant of WP (each adjusted to 3.5 × 10 ^8^ platelets/mL) after platelet preparation (basal) and after incubation for 30 minutes with buffer (control), for 30 minutes with 15 µg/mL Pam3CSK4 or for 30 minutes with 15 µg/mL LPS. Results are presented as mean ± SEM; *n* = 10; * *p* < 0.05, as indicated; # *p* < 0.05, LPS- and Pam3CSK4-stimulated samples of WP compared with corresponding samples of PRP. ∼ *p* < 0.05, TRAP-6- or thrombin-stimulated values compared with corresponding basal values; ° *p* < 0.05, thrombin-stimulated samples of WP compared with corresponding TRAP-6-stimulated samples of PRP. The histograms show the mean fluorescence intensity (MFI) of CD62P expression ( **F** ) and of fibrinogen binding ( **G** ) in freshly prepared PRP and WP suspensions, without stimulation (basal) or after stimulation with 10 µM TRAP-6 for 2 minutes. Isotype controls indicate unspecific binding. The data are presented as mean ± SEM; *n* = 10; * *p* < 0.05 (TRAP-6 stimulated values compared with basal values); ** *p* < 0.05 (basal values of WP compared with basal values of PRP).

The concentrations of these chemokines were measured in PRP and in the medium of WP after incubation for 30 minutes with 15 µg/mL Pam3CSK4 or with 15 µg/mL LPS ( Fig. 4 ), as threshold concentrations inducing or supporting aggregation in WP ( Fig. 1 ). Basal chemokine concentrations in PRP were significantly lower than in the medium of WP, except for sCD40L with comparable values. As controls without TLR stimulation, the basal values were determined after 30 minutes of incubation with buffer. The values for RANTES, PDGF, and PF4 increased significantly and were higher in WP than in PRP ( Fig. 4A–C ). The NAP-2 concentration increased significantly in PRP only, reaching the level of the WP medium ( Fig. 4D ). In contrast, sCD40L levels remained stable in PRP and increased in WP ( Fig. 4E ).

Upon stimulation with Pam3CSK4 or LPS, the levels of RANTES, PDGF, and PF4 rose significantly, more emphasized in WP compared with PRP ( Fig. 4A–C ).

In PRP, neither Pam3CSK4 nor LPS induced higher levels for NAP-2 or sCD40L ( Fig. 4D, E ). However, in Pam3CSK4- or LPS-stimulated WP, both chemokines were increased, reaching significantly higher levels than in stimulated PRP samples.

Compared with stimulation with 15 µg/mL Pam3CSK4 or LPS, the chemokine concentrations reached much higher levels upon strong platelet activation with TRAP-6 or thrombin ( Fig. 4A–E ). The values were higher in PRP for RANTES and PDGF, and for PF4 and NAP-2 in a similar range. A remarkable difference was observed for sCD40L with diminished levels in PRP compared with WP.

The activation levels of WP and PRP were measured by flow cytometric analysis of CD62P expression ( Fig. 4F ) and fibrinogen binding ( Fig. 4G ). The basal values were approximately twofold higher in WP suspensions compared with PRP for both parameters. However, responsiveness of platelets was well maintained upon TRAP-6 stimulation, reaching manifold elevations of CD62P expression and of fibrinogen binding, with comparable levels in PRP and WP.

## Discussion

There are several studies dedicated to TLR expression and function in platelets. However, many results are hardly comparable since some of the studies investigated WP, 5 8 15 whereas others were performed with PRP. 4 18 29 As a consequence, we intended to focus on the comparative analysis of TLR2- and TLR4-mediated platelet function, receptor expression, and related secretory characteristics in both milieus, PRP and WP.

According to previous studies, 16 neither Pam3CSK4 as TLR2 agonist nor LPS as TLR4 agonist was able to induce platelet aggregation in PRP. In WP, 15 µg/mL Pam3CSK4 induced stable aggregation responses, essentially confirming the results of former studies using 10 µg/mL Pam3CSK4. 7 8 In contrast, LPS alone (even up to 100 µg/mL) did not induce platelet aggregation in WP, but it promoted aggregation by simultaneous stimulation with threshold concentrations of thrombin, as described by Rivadeneyra et al. 15 In conclusion, both receptors, TLR2 and TLR4, are involved in mechanisms leading to platelet activation and aggregation, 1 albeit attenuated in the milieu of PRP compared with WP.

The elevation of receptors, in WP and PRP, was more pronounced with potent platelet agonists like convulxin and TRAP-6 than with weak agonists like ADP or epinephrine. This upregulation of TLR2 and TLR4 expression is similar to other ligands or receptors (e.g., to CD62P), which are recruited from platelet α-granules to the platelet surface upon agonist stimulation. The washing procedure contributes to slight preactivation of platelets, indicated by higher levels of CD62P and fibrinogen binding in comparison to PRP, and in that way, presumably contributing to higher TLR2 and TLR4 expression levels in WP. According to our results, an increase in TLR4 levels was reported in thrombin-stimulated WP by Tsai et al, 30 but not by Aslam et al for TLR2 and TLR4. 10 In activated platelets from platelet concentrates, an elevation of TLR2 expression was observed, 4 in contrast to falling TLR4 levels. Another study did not reveal significant effects of 15 µM TRAP on TLR2 and TLR4 expression 14 in PRP, but remarked variable individual responses as an explanation for conflicting results in addition to heterogeneous experimental conditions.

In general, it is well known that agonist-stimulated platelets release several immunomodulating ligands and cytokines like sCD40L, PF4, RANTES, PDGF, or NAP-2. 31 32 The ability of platelets to shed some contents of α-granules upon TLR stimulation had also been demonstrated in previous studies, 1 22 29 but quantification has frequently been performed in the supernatant of PRP. 1 18 29

As the lowest concentrations promoting platelet aggregation, 15 µg/mL Pam3CSK4 and 15 µg/mL LPS were able to induce the shedding of all investigated chemokines from WP (approximately two- to fivefold compared with unstimulated controls), supporting the results of aggregation studies with the potential of TLR2 and TLR4 for platelet activation. In this context, the capacity of TLR2 and TLR4 to mediate the release of NAP-2 has been shown for the first time. Compared with strong agonists like thrombin or TRAP-6, the secretory effects were submaximal and more pronounced with the TLR2 ligand Pam3CSK4. In contrast, in PRP, the induced increment was smaller (∼1.3- to 2-fold), the peak concentrations of chemokines were lower than in WP, and there was no increase of NAP-2 and sCD40L. Such a remarkable difference for sCD40L was observable even after maximal platelet activation with TRAP-6 or thrombin.

In consequence, plasma factors should be considered as relevant modulators of chemokine levels (e.g., interfering cleavage by endopeptidases 33 or protein binding 34 ). In addition, the higher degree of preactivation in WP, resulting in pronounced cellular responses, is another physiological explanation for the increased release of chemokines compared with PRP. TLR4-mediated degranulation of platelets has also been quantified in a recent study using PRP, showing heterogenous results with unaffected (PF4), decreasing (RANTES, PDGF), or slightly increasing (sCD40L) chemokine levels under stimulation with 3 µM LPS. 18 Furthermore for TLR2, it was reported that high doses of Pam3CSK4 (100 µM) have led to significant releases of PF4 or RANTES and less markedly of sCD40L in PRP. 11 Instead, in another study with WP, the shedding of von Willebrand factor was detectable even with low doses of 1 µM Pam3CSK4 or 1 µM LPS. 15 Similar to our findings, Rex et al could show by immunoblotting that 10 µg/mL Pam3CSK4 is able to induce secretion of PF4 from WP, more intensively than under ADP stimulation and to a lesser extent than with 0.5 U/mL thrombin. 8

As a limitation of the study, it must be considered that expression patterns and secretory responses may be influenced variably by different procedures for washing platelets or by different storage media. However, methods and conditions in this study have been commonly used for experimental platelet research. 35 36 Furthermore, basic results (e.g., the measured concentrations of secreted chemokines) have been in the range of published data indicating reliable results. Due to the variable release patterns, the degranulation of certain chemokines (e.g., NAP-2) may be driven by specific processes initiated upon TLR stimulation, possibly dependent on the type of agonist, as observed for the stimulation of other receptors. 37 In these experiments, Pam3CSK4 and LPS (from *E. coli* ) were used for stimulation, but future studies should confirm the findings by additional TLR2 and TLR4 ligands. Obviously, TLR2 exerts its effects via the phosphoinositide 3-kinase (PI3-K)/Akt pathway 7 and PAR-1 dependent NF-κB phosphorylation. 11 In this context, it would be of interest to analyze further receptor systems and signaling pathways associated with TLR stimulation.

This direct comparison of WP and PRP has rendered several novel contributions to the understanding of TLR2- and TLR4-mediated platelet responsiveness. The dependence of platelet preparation on TLR integrity is an important issue for the design of experimental settings in basic research. In addition, the results are of clinical relevance (e.g., for the optimization of storage milieus in transfusion medicine). Plasma reduction is discussed to prevent adverse transfusion reactions by removing accumulated cytokines. 38 The modulation of the plasma content may also support the preservation of TLR-dependent platelet function.

In summary, platelet activation results in a rapid and sustained increase of TLR2 and TLR4 expression in both milieus. In WP, but obviously not in PRP, receptor stimulation can induce (TLR2) or support (TLR4) platelet aggregation. In WP, TLR2 and TLR4 stimulation promotes the subtotal release of chemokines from α-granules, shown for NAP-2 for the first time. In PRP, the secretory effects are less pronounced for RANTES, PDGF, or PF4, and not detectable for NAP-2 or sCD40L.
